# “Why should I have come here?” - a qualitative investigation of migration reasons and experiences of health workers from sub-Saharan Africa in Austria

**DOI:** 10.1186/s12913-015-0737-z

**Published:** 2015-02-26

**Authors:** Elena Jirovsky, Kathryn Hoffmann, Manfred Maier, Ruth Kutalek

**Affiliations:** Unit Ethnomedicine and International Health, Department of General Practice and Family Medicine, Centre for Public Health, Medical University of Vienna, Vienna, Austria; Department of General Practice and Family Medicine, Centre for Public Health, Medical University of Vienna, Vienna, Austria

**Keywords:** Health worker migration, Sub-Saharan Africa, Austria, Qualitative research, Migration reasons, Migration experiences

## Abstract

**Background:**

There are many health professionals from abroad working in the European Union and in Austria. The situation of sub-Saharan health workers in particular has now been studied for the first time. The objective was to explore their reasons for migration to Austria, as well as their personal experiences concerning the living and working situation in Austria.

**Methods:**

We conducted semi-structured, qualitative interviews with African health workers. They were approached via professional networks and a snowball system. The interviews were transcribed and analysed using atlas.ti.

**Results:**

For most of our participants, the decision to migrate was not professional but situation dependent. Austria was not their first choice as a destination country. Several study participants left their countries to improve their overall working situation. The main motivation for migrating to Austria was partnership with an Austrian citizen. Other immigrants were refugees. Most of the immigrants found the accreditation process to work as a health professional to be difficult and hindering. This resulted in some participants not being able to work in their profession, while others were successful in their profession or in related fields. There have been experiences of discrimination, but also positive support.

**Conclusions:**

Austria is not an explicit target country for health workers from sub-Saharan Africa. Most of the study participants experienced bad work and study conditions in their home countries, but they are in Austria mostly because of personal connections. The competencies of those who are here are not fully utilised. The major reason is Austria’s current resident and work permit regulations concerning African citizens. In addition, the accreditation process and the German language appear to be barriers.

## Background

Up until the 1970s, health workers from a small number of sub-Saharan African countries migrated to a limited number of high-income countries [1]. However, today the picture has changed: sub-Saharan African countries experience an ever increasing out-flow of much-needed health workers to a growing number of destination countries [2]. This has led to a “global health workforce crisis” [3]. According to the World Health Organization (WHO), there is a worldwide deficit of 4.3 million health workers; in Africa alone there is a shortfall of 1.8 million, and this is affecting access to basic health care [3].

Different push- and pull-factors have been identified to explain why so many health workers leave their home countries: known push-factors are the lack of opportunity for professional development, lack of availability of equipment and supplies, heavy workload, low wages, low job satisfaction, and the threat of political instability and conflict. Pull-factors include better remuneration, better working conditions, and opportunities for professional development in target countries [4-7]. Current studies continue to confirm these factors [8].

One cause of these migration movements is the strategy of high-income countries to recruit health workers from developing countries [9]. In addition, in several source countries, doctors are encouraged to look for opportunities abroad; there is a “well developed culture of medical migration” [10]. The United Kingdom (UK), United States (US), and Canada attract health workers migrating from sub-Saharan Africa, and all have existing or historic active recruitment policies [2]. In the UK, 37% of all registered doctors have been trained overseas: more than half are from Africa or India [11]. Such recruitment policy has been subject to ethical debate and challenge since the 1970s [12]. This debate led the WHO to develop the voluntary Global Code of Practice on the International Recruitment of Health Personnel in 2010. The code provides ethical guidelines for recruitment to protect and strengthen health systems in low-income countries, those in economic transition, and small island states [13].

Several studies have explored the reasons why health workers migrate from Africa [4,5,10,14-16]. Many studies have focused on the intention to migrate, rather than on actual reasons for migration. Only some have included African health workers who are now living overseas [11,17]. Recently, studies have been conducted, for instance, on the retention of migrant health workers in Ireland, or their professional experiences there [18,19]. Up until now, the situation of health workers from sub-Saharan Africa in Austria has never been studied. Such a study is important because Austria does not have a policy of active recruitment. The Austrian study is linked to others conducted in Europe (Belgium, UK) and Africa (Botswana, South Africa) in the same period, in the same larger context within the larger framework of the HURAPRIM project (EU-FP7) on human resources in primary health-care in sub-Saharan Africa (http://www.huraprim.ugent.be/). The overall aim of all of these studies was to understand why health workers from different regions of sub-Saharan Africa leave their countries of origin [8]. This paper in particular focuses on the reasons for migration, and the post-migration experiences of health workers who migrated from a sub-Saharan African country to Austria.

### Austrian migration policy

Austria, a country of 8.49 million people, is part of the Schengen Area comprising 26 European countries that have abolished passport and any other type of border control at their common borders [20]. EU citizens and European Economic Area (EEA) nationals are visa-exempt and legally entitled to enter and reside in each other’s countries. Ireland and the United Kingdom operate their own separate visa policies, as do certain overseas territories of EU and EEA member states [21]. There are no bilateral agreements between African countries and Austria or other Schengen countries. Citizens of an African country need a visa to enter Austrian territory.

Employment in Austria is subject to both a work permit and a residence permit. Before a work permit applicant's departure to Austria, a residence permit has to be obtained. Neither of these permits can be granted while being in Austria on a tourist visa. With the exemption of scientists and researchers with assignments at universities and certified research institutions, all citizens of non-EU countries need a work permit. A prospective employer needs to file an application at Public Employment Service Austria [22]. In addition, since 2011, the immigration of international skilled workers interested in relocating to Austria has been regulated by a criteria-led immigration system that allows qualified workers from third countries (other than EU member states, such as Iceland, Liechtenstein, Norway and Switzerland) and their family members to permanently immigrate to Austria. The points system is based on qualifications, work experience, (German) language skills, age, and studies completed in Austria (for those who are very highly qualified). In contrast to less qualified applicants, higher qualified immigrants do not need to prove German language skills before migration [23].

Foreigners who are not either married to an Austrian citizen or who are descendants of a new citizen, can apply for citizenship after at least 10 years of permanent residence in Austria. They have to prove that they have a secure income and no criminal record, and show German language proficiency as well as a knowledge of Austrian history and current affairs [23].

Health workers are not allowed to work in Austria unless they have German language skills and nostrification (nostrification is the process by which the Austrian government grants accreditation to a diploma). Criteria for the nostrification are the content, range, and formal requirements equivalent to the respective Austrian degree [24]. Citizens of EU member states do not have to nostrificate their diplomas and do not require work permits [25].

### The Austrian health system

Austria’s health system is a Bismarck system. Health services are financially covered by public health insurance companies. The system is divided into ambulatory and hospital sectors; an Austrian peculiarity is that specialists can work in both sectors. The focus is on hospital care with no gatekeeping: patients can, in fact, choose their level of care; they have free access to nearly every specialist or hospital. The country has one of the highest hospital densities and hospital admission rates in the EU. In relation to health outcomes, general life expectancy is high, although, in comparison with other EU countries, the healthy life expectancy is below average [26,27]. Austrian regulations concerning care professions are peculiar in comparison to, for instance, Sweden and the UK, as there is no specific training for the few nurses working in the ambulatory sector. Nurses are not allowed to triage, diagnose, or provide treatment on their own. They can only act upon delegation by doctors. They are, therefore, always dependent on doctors, and their work is often reduced to activities like washing or mobilizing patients. They have performance responsibility and not commanding responsibility [28]. This is an Austrian peculiarity, which easily leads to a deskilling of nurses migrating to Austria from other countries, especially if people come from countries where nurses have a broader range of responsibilities.

### Health work force in Austria

Austria has one of the highest numbers of medical graduates in the OECD. It is a net-exporter of doctors; more doctors are trained than are retained in the country [29]. In recent years, between 1,100 and 1,200 doctors per annum were newly registered in Austria. The proportion of newly registered foreign-degree holders in 2008 was just 4% (40 of a total 1132 doctors). These foreign-degree holders mostly hold German degrees, and figures therefore include Austrians who have completed their studies in Germany.

Currently, in Austria, 28% of foreign doctors are from EU countries [26]. In 2004, 6% of the personnel working in hospitals were foreigners. Sixty-six percent were EU nationals and 34% third-country nationals [30]. The main source countries are Germany, Switzerland, and South Tyrol, an Italian region with a German-speaking minority, but more and more migrant doctors also come from Arabic countries. From the new EU member states, most immigrating doctors come from Hungary, the Czech Republic, and Slovakia [25]. The last free market restrictions imposed by Austria and Germany on the new member states which joined in 2004, were lifted in 2011 [31]. There are also significant numbers of illegal foreign health workers working in homecare in Austria, mainly from Slovakia [25]. Austria especially “imports” dentists, with the majority of foreign dentists coming from Germany or Hungary. In recent years, more than 40% of newly registered dentists have held foreign diplomas [26].

Germany and Switzerland are the most common destination countries for Austrian doctors emigrating to work abroad, particularly in the hospital sector [26]. There is growing concern that this trend has potential risks for the Austrian health care system [26]. Austria, along with many European countries, very likely faces a shortage in general practitioners, particularly in rural areas, in the near future [27,29].

Furthermore, there is a clear shortage of care staff in Austria, which is problematic especially in long-term care. Therefore, Austria has long been a net importer of nurses [26]. For instance, there is a long history of recruiting nurses from the Philippines [32]. The nursing profession is not very highly esteemed in Austria and nurses are overworked and underpaid [33,34], a fact that does not encourage enough people to choose this profession.

Recruitment agencies actively recruit health workers, and especially care staff, from Austria’s neighbouring countries, in particular, the new EU member states Hungary, the Czech Republic, and Slovakia [25]. However, there is no evidence that there is active recruitment of health workers from Africa. This makes it possible to conclude that in contrast to other European countries that have a large number of health workers from sub-Saharan Africa [11], there are relatively few in Austria. Reasons for this seem to be the language [35] and the difficulties in the accreditation of diplomas and of tertiary education in general.

### Estimates of the number of migrant health worker from sub-Saharan Africa in Austria

The number of migrant health workers from sub-Saharan Africa does not appear as a particular category in official statistics on the Austrian health work force, as shown above. Therefore, we can only estimate their numbers based on available information. There are no data available on how many people who trained as health workers in a sub-Saharan country actually work in their profession in Austria, and how many either never received an accreditation or never attempted to get one. This means, from the available numbers alone, we cannot determine how many of these health workers are consistent with the study participant criteria of our study: that is, of being born and educated in Africa.

Overall, compared to other groups of immigrants, people from Africa are few [36]. People with foreign heritage represent 11.5% of the Austrian population or 970,500 people, whereas approximately 23,200 people or 2.4% of foreigners are from Africa, including North Africa [36,37]. There is no specific data available on their respective education or profession. According to information provided by the Austrian Medical Chamber, there are more than 40,000 doctors (excluding dentists) actively working in Austria; the ratio is 431.5 doctors per 100,000 people. Of the total number of 4,410 foreign doctors (excluding dentists) who accredited their foreign-diploma in Austria in 2011, 18 were citizens of sub-Saharan African countries. Numbers of doctors from Africa who have not been accredited in Austria or who are just about to go through this process are not available. Furthermore, there is no information on the numbers of doctors from sub-Saharan Africa actually working in Austria. In 2011, the foreign nurse workforce in Austria was 6.7%, of which 1.7% are from countries including sub-Saharan African countries [38].

In this present study, we aimed to identify the reasons for the migration of health workers from sub-Saharan Africa currently living in Austria. Furthermore, we aimed to document these health workers’ particular migration experiences. Neither phenomenon has been studied before. For this purpose, we conducted qualitative semi-structured interviews. Given the above-discussed context, we on one hand illustrate why sub-Saharan African health workers still come to Austria, a country with no recruitment history in sub-Saharan African countries. On the other hand, we discuss our results in terms of the worldwide challenge of health worker migration, and provide explanations for some of the interesting findings presented here. We present our findings in the international context of global health worker migration. Our results are interesting for countries with a similar migration policy and without active recruitment from sub-Saharan Africa.

## Methods

### Semi-structured interviews

Within the framework of the above-mentioned HURAPRIM project, a project targeting the working situations of health workers in sub-Saharan African primary health care, we conducted semi-structured interviews. The use of semi-structured interviews makes interviews comparable, as the same set of questions is asked of multiple participants [39]. While repeatedly addressing specific aspects of a research question, semi-structured interviews allow the participants to freely narrate [40]. The interview method was chosen for the HURAPRIM project because of this capacity. Interviews with migrant health workers from sub-Saharan Africa conducted in different partner countries were comparable due to the use of the same interview guidelines.

The interview guide included questions regarding migration motives, views on primary health care, changes needed in the country of origin to retain health workers, transnational ties of the migrant health worker, and future plans [8,41]. For the Austrian research, the English guideline was translated into German and French; interviews were conducted in the language preferred by the study participant.

The interview phase of this qualitative study lasted from July 2011 until April 2012. Qualitative research methods were employed to get an in-depth insight into the personal experiences and motivations of health workers. We aimed to interview as many health workers from Africa as possible.

### Recruitment strategy

Study participants had to fulfil the following criteria: they had to be born in sub-Saharan Africa and should have completed at least part of their health-education in the country of origin or any other African country. With these criteria, we aimed to include people who had first-hand living experiences in a sub-Saharan African country, as well as insights into the local health system, at least from an educational perspective. Altogether, we approached more than 150 contacts via e-mail and telephone, and we asked around in our personal and professional networks. The e-mail addresses we used were those available on homepages of health-care providers and those we received via the aforementioned networks. In this manner, we contacted individual health workers, migrant organizations, social organizations, employers of all public hospitals and laboratories, as well as health worker educational facilities in all of Austria’s nine provinces. Furthermore, we contacted umbrella organizations such as the Austrian Medical Chamber (ÖÄK), the Austrian Nurses Association (ÖGKV), and the Vienna branch of the International Organisation of Migration (IOM), as well as several informal networks of the migrant population in Austria. In addition, a snowball sampling method was applied by asking our study participants for further contacts [42,43]. The decision whether or not to contact us had to be made by the individual health worker himself or herself, because Austria has strong data privacy regulations and umbrella organizations can only forward a research request to their members or employees.

Recruitment proved to be difficult. From several African migrant associations we received feedback that there is a certain frustration among African people in Austria. Representatives from these organisations told us that researchers from various disciplines contact them about research objectives related to Africans in Austria. Persons responsible in the organisations indicated that “Africans” in Austria feel tired of – and even abused by – being research subjects. As these persons responsible were important gatekeepers, their view most likely had an important impact on the sample returns.

Despite our best efforts to recruit participants, due to time and funding restrictions, we concluded our search for further participants in April 2012. Eventually, we conducted ten interviews.

All interviews were recorded with a digital recording device and additional notes were made during and after the conversation. Only the study participant and the researcher (EJ), who is an experienced qualitative interviewer, were present. As chosen by the study participants, the interviews were conducted in the workplaces of either the interviewer or the study participants. One interview took place in a café. Each interview took between 30 and 90 minutes.

### Analysis method

The interviews were transcribed verbatim after conducting all interviews. Two team members did preliminary coding on paper to collect first themes after the interviewing process was concluded. Subsequently, the same researcher who conducted the interviews made a more detailed thematic analysis using atlas.ti. Coding was done deductively [44]: categories and codes were built on topics that appeared on the basis of the first revision of the material as well as the structure postulated by the interview guidelines.

### Informed consent and confidentiality

Before the interviews, all study participants were informed about the HURAPRIM project and its objectives, as well as the purpose of the interviews. Written consent was obtained from all study participants. No incentives to participate were offered. The ethics committee of the Medical University of Vienna approved the study (EK-Nr: 989/2011). We guaranteed our respondents confidentiality to create a safe interview environment. The respondents’ anonymity is necessary to avoid any potential negative consequences for them. In particular, concealing formulations in publications serve this purpose [45]. As the sample is very small, for instance, we do not provide information on the respective countries of origin of our respondents in this paper.

## Results

### Coming to Austria by chance

To study the issue of health worker migration from sub-Saharan Africa in the Austrian context, we conducted semi-structured interviews with seven doctors and three nurses. Our study participants come from different sub-Saharan African countries and have diverse professional backgrounds. Table 1 shows that four male and six female participants took part in our study. Four of our study participants do not have Austrian citizenship, one has had it since 2011, and the other five had it for between 10 and twenty years. The nurse and the doctor with Austrian citizenship who finished their education in Austria (P 4; P 8) would not fall into the category of foreign health worker in the statistics mentioned above. Three male participants were trained doctors before migration (P 1; P 2; P 7), and two are still working in their profession (P 2; P 7). Three female participants were trained doctors (P 3; P 4; P 5), and another one had almost finished her medical education (P 10). She and another one of the three trained female doctors are now working in different professions (P 4; P 10). Three nurses – one of them male – still work as nurses (P 6; P 8; P 9).

**Table 1:** **The study participants**

	**Gender**	**Age cluster**	**Citizenship**	**Private situation**	**Education**	**Current profession**	**Paramount motivation**
P 1	M	30-40	Other (Africa)	Married; children	Doctor	Different Occupation	Partnership
P 2	M	50-60	Other (EU)	Single	Doctor	Doctor	Education possibilities; Return impossible due to continuing unstable political situation
P 3	F	40-50	Austria	Married; children	Doctor	Doctor	Education possibilities; Partnership
P 4	F	40-50	Austria	Single	Doctor	Different Occupation	Return impossible due to continuing unstable political situation
P 5	F	30-40	Austria	Married; children	Doctor	Doctor	Partnership
P 6	F	50-60	Other (EU)	Married; children	Nurse/Midwife	Nurse	Partnership
P 7	M	50-60	Austria	Married; children	Doctor	Doctor	Education possibility; family in Austria
P 8	F	30-40	Austria	Married; children	Nurse	Nurse	Refugee due to war
P 9	M	30-40	Other (Africa)	Single	Nurse	Nurse	Better job opportunities and better payment
P 10	F	30-40	Other (Africa)	Married; children	Medical Student	Different Occupation	Partnership; political situation

Seven of our 10 study participants – four doctors (P 1; P 3; P 5; P 7), a medical student (P 10), and a nurse (P 8) –, are now married and have children. A doctor (P 7), who was single when he started his coursework in Austria, found that being singly made it easier for him to get ahead professionally. A nurse (P 8) was also single, as she was quite young when she immigrated. Two male participants, a doctor and a nurse, stayed single (P 2; P 9). A female former doctor also remained single (P 4). She migrated with her mother, with whom she still lives.

Only one of our study participants, a nurse, had actively planned to come to Austria. His main reasons for migration were the better job opportunities in Europe as well as better and regular payment. His aunt, who migrated to Europe (but not to Austria) before he did, convinced him to leave (P 9). His reason for leaving his country was that he had difficulty finding a good job in his home country. Had he been able to find work, he would have remained in his home country:

It is only the political and governmental problems that are the reasons for so many to come here. […] I was eighteen when I came here, this means if I had a fix[ed] position at this time, why should I have come here? Because, the first two years that I have spent with my German course, I would have done something there already. (P 9)

Another study participant, a doctor (P 7), has a different migration history than the other eight study participants: his main reason to migrate was to secure better working conditions and continue his specialist education. In his first job in an emergency unit in his home country, he found the working conditions unsatisfactory. He already had relatives in Austria, which made moving more attractive:

If I do something and cannot do it right, I [would] rather do not do it at all. In the country in the 1990s, it was so shattered; everywhere was suffering, in the hospitals was nothing. […] It was impossible to do medicine there. It was clear to me, that I cannot do it for long. (P 7)

Coming to Austria was his chance to fulfill his dreams:

I had the possibility to do what I have dreamt of, I took it, I did not let go and I carried on. Afterwards I did the nostrification and continued with my education. This was just wonderful. I was alone, not married, no kids. This was the chance for me and I took it. (P 7)

Another doctor (P 3) came to Europe initially to study; better education opportunities were, therefore, also her reason to leave Africa. She returned to Africa and had already started practicing in sub-Saharan Africa but finally came back to Austria for the sake of her marriage to an Austrian citizen (P 3). Her reason to move to Austria was, therefore, personal and not job-related. She did not initially plan to come to Austria.

The other seven study participants also did not initially plan to move to Austria. Two were refugees: a doctor from an Anglophone country who would have preferred to go to another English-speaking country accepted that the political situation in her home country meant she could not return. At the time, she was completing an internship in North Africa, but she could not stay there, either. Eventually she ended up in Austria:

So we decide, we weren’t able to stay in [location of internship], at the same time it was very difficult to return back [home]. My father and my brother were arrested, they are in jail, we are not able to live, and there is no secure life there. Then I decided to go somewhere where I can find better life for us, and yeah, with the help of some friends I managed to escape and to come here. I didn't plan to come to Austria because I would have preferred to go to somewhere where I can easily learn the language. My colleagues or my friends are already in London or America; it just took them two years to study the language very intensively, and then it was easier. However, here, with a totally new language, for me it was hard. But, I think it was my luck, I told you, far in the beginning, it was my luck to be here because I found my chance here, in comparison to my other friends. (P 4)

The other refugee left her home country because of war; her family had been persecuted and her parents killed. After having fled to several different countries in Africa, she came to Austria, where one of her brothers already lived, and again took up her nursing education (P 8). These two cases seem to be exceptional in comparison to the other migration stories. Neither of these two participants chose to come to Austria for personal/private reasons, nor did they come for professional reasons. They had to make the best of the situation when they ended up in Austria.

The other five study participants engaged in relationships which finally led them to follow their partners to Austria: a nurse had already left her home country more than thirty years earlier and moved to a country neighbouring Austria, where she met her husband, with whom she then moved to Austria (P 6). A medical student married someone from her home country who had already been living in Austria for several years. She abandoned her education and was not able to continue it in Austria (P 10).

Another medical student met his wife, an Austrian, while she was doing an internship in Africa. He is about to learn German and meanwhile continues his education in a health-related field (P 1). Another study participant had already been part of the internal brain drain in her country of origin, as she was working on an international epidemiological research project. During that project, she met the Austrian man who became her husband. He had been doing an internship in Africa. Therefore, she moved to Austria with him:

I did not want to work in a public hospital; this was clear to me. [With the work offer] I preferred working in a laboratory, to be able to work. This was my decision; this was why I went into research. I did not want to work in public hospitals because the conditions were so bad; I experienced so many bad things. (P 5)

Our study participant (P 5) had been professionally successful and left Africa for private rather than professional reasons. However, it is interesting that in her home country she has chosen to work in a more attractive research environment rather than, for instance, in basic health services. She had negative experiences during her training, which led her to decide not to seek such employment.

In summary, the results showed that for most of our study participants, the decision to migrate was not a professional one, but rather situation-dependent. Even though they had had bad experiences in their workplaces, they originally did not intend to migrate.

However, whatever their reasons/motivations for coming, whether by chance or intentionally, the outcome was the same: they had to follow the same steps to continue in their careers, once they reached Austria. Those steps often presented a new set of barriers.

### Accreditation process - a barrier

The accreditation process necessary to be health worker in Austria shaped the experiences of our study participants. The results show that three of our study participants spoke about this process positively: one was already partly educated in Europe; she did not have any problems with accreditation and recently opened her own medical office in a major Austrian city (P 3). The nurse who had been working in Europe for 17 years before coming here only had to take some courses about Austrian legislation (P 6).

As mentioned above, one of our study participants anticipated before he came to Austria that he had to go through an accreditation process in order to practice there. The training in Austria was challenging. In particular, the new language he had to master complicated the situation. However, he acknowledged, the hard training in his home country prepared him for the difficulties that he encountered abroad:

The education here was not easy, but I always say that whoever manages to get through nursing education in my home country can get through it anywhere in the world. (P 9)

For the others, the process was and still is difficult: they had to repeat their internships or even their whole education, which often took several years (P 5; P 8; P 9). One study participant plans to get his medical degree accredited in Ireland because with his Anglophone background, it will be easier. He plans to go through the accreditation process in Austria afterwards (P 1). It thus seems as if a “work-around” is possible due to Austria’s membership in the EU.

Another study participant originally planned to continue her education when her children were older, but is still not in a position to do so. Continuing her education would clearly take too much effort (P 10):

Normally, as I informed myself, I would need to legalize all my papers there, bring them here to show what I have accomplished in my education and then I would be able to finish my studies here. However, here with my family I just do not know how I could manage. At home, it would be ok; I would have someone to take care of the children. But here, I have to do everything alone: children, appointments, and school, surviving… (P 10)

As with the above participant, P 4 also found it impossible to repeat her education in order to take a new job. Had these two workers remained in their home countries, they would have found employment. However, by migrating, their potential is not fully realized.

### Ambivalent experiences in new personal and professional situations

The inability to work in one’s chosen health profession can lead to frustration, as our results show. Immigrants who are not able to continue in their professions (P 10; P 4; P 1) feel that they cannot fully live up to their potential. This is difficult emotionally, as it can be very damaging to one’s confidence. These health workers perceive their own social status as lower, because they are not working up to the level of their education. One of our participants is working in a profession that is not related to health care or medicine at all, and she is the most frustrated (P 10). She feels sad and misses her former occupation whenever she has to go to a hospital in Austria:

I have to [finish my education]! If I don’t do it, it is like I was ill. It makes me sick when I go to the hospital, I feel sad. I think about that I should be here and help the patients! That I could work here. I miss it all the time. (P 10)

In another part of the interview, she argued that it would nonetheless be difficult for her to continue because she lacks the supportive social network of her family.

The other participants have been able to find satisfying professions where they can use their knowledge, even though they are not able to practice medicine (P 1; P 4). In particular, the social worker (P 4) stressed that she found her new profession morally and socially relevant, which compensates for the fact that she cannot practice medicine. One other study participant (P 6) had been trained as midwife and as a nurse (a combination common in her country of origin). She finds it disheartening that in Austria she is not allowed to work as both. However, she is satisfied with her career – she is the head nurse of a hospital ward – and is proud to meanwhile hold this leading position.

Two women talked about how frustrating it was to have been prevented from working in their chosen profession while they waited for accreditation and to have been relegated to roles as homemakers (P 10; P 5).

Two young men had to work in the low-paying sector in the beginning of their stay in Austria in order to be able to afford their language lessons and living expenses (P 7; P 9).

Many considered the education they received in Austria less demanding than what they experienced in their home countries (P 5; P 6; P 9). The education in the home country was perceived as much more thorough and challenging (P 6). A doctor (P 5) felt that, in Austria, she learned new things, for instance, to work with diagnostic tools such as a CT scanner. In addition, the study circumstances were perceived as generally better, particularly the libraries (P 7). However, unlike in sub-Saharan Africa, the students in Austria had to do a lot of paperwork, which prevented them from coming into contact with the patients and learning in a clinical practice:

Well, here, sometimes, we did not learn much during rounds. Back home, the professor always taught during rounds. Students came along, we actually saw the patient in his bed, and he did “bed teaching.” During my internship, it was really “bed teaching.” But in [major Austrian city], we are more there as staff. I found that we were responsible for the referrals, for the administrative work. Sometimes we could not take part in the rounds, because we had to write so many letters. (P 5)

Also, another doctor (P 3) said that the education in her home country was more hands-on than the one in Austria, a fact she perceived as beneficial. A positive aspect for a nurse (P 8) was that the education in Austria was free, in contrast to her home country, where it was expensive.

Most of our study participants implied that they experienced discrimination or racism in Austria, not from co-workers but from patients (P 8), examiners (P 6), or in daily life (P 7). Experiences of sexism were neither implicitly nor explicitly addressed. The participants said that they had heard, before coming to Austria, that Europeans have negative attitudes about Africans. They hinted that these expectations were substantiated. One nurse said that her colleagues had to convince patients to allow her to care for them (P 8). A nurse said that she felt degraded because her expertise as a health worker was belittled when she applied for accreditation (P 6). She felt that this was a result of her being from Africa. Another nurse (P 9) said that when he applied for advanced education, and the secretary in charge of registration assumed he was applying as a hospital porter. There is no doubt that what he experienced was racism:

It was funny, because I went to this school, to the secretary and told her that I wanted to do the education for psychiatric care. Then she took out some forms. And I said, “But this is the form for a hospital porter.” And she said, “But, what do you want?” And I said, “Well, I have a diploma; I am already a general [nurse].” Well, she told me that I have to go to the head. Immediately I had my interview, my motivational talk, with the head. He asked me where I came from and I said from [country of origin]. [He said,] “Oh, I see, you are already a graduate.” I said, “yes I have worked here for four years.” [He said,] “Yes, very well, come here and bring your papers, your nostrification, your diploma…” And so, I got the place for psychiatry. (P 9)

Several study participants mentioned initial problems common to being a newcomer in a foreign country (P 8; P 5) but all of them overcame their early difficulties and are now professionally successful. However, not all were able to stay in a health care profession, as mentioned above. In the beginning of their stay in Austria, our study participants also found support either from teachers and colleagues (P 7; P 9), or through private networks of a spouse (P 1). One participant said that others were sympathetic to her being a newcomer and a refugee (P 8).

Coming to a different country was challenging, as most of our respondents anticipated before leaving their home countries. Nevertheless, for all our respondents, immigration was a mixed experience with both positive and negative elements.

## Discussion

The aim of this study was to examine the reasons that sub-Saharan health workers in Austria migrated, and to illustrate their experiences, as Austria is a country that has never actively recruited in in sub-Saharan Africa. This is the first study of its kind. It is of interest to countries with a migration policy similar to that in Austria and without active recruitment from sub-Saharan Africa.

The study contributes to the understanding of the dynamics of health worker migration to Europe. Furthermore, it adds to a better understanding of some of the experiences of migrant health workers in European countries, and is relevant in context of the global health worker migration and the lack of human resources, particularly in Africa [46].

Austria is not an explicit target country for health workers from sub-Saharan Africa and the number of such workers in Austria is low. There is a human resource crisis in Africa, but Austria is not actively partaking in any recruitment programs to worsen the problem there. Nevertheless, Austria recruits health workers from eastern member states of the EU [25,26,47] and might soon struggle with brain drain itself [48].

The active recruitment of health workers from Africa, as well as their migration to Europe and Austria, is complicated by several factors: first, the diplomas from non-European Union countries are not easily accredited. Second, every employee who gets a work permit in Austria needs to prove certain German language skills. Third, African citizens in general do not easily obtain long-term residence or work permits for the Schengen Area. There are no bilateral agreements between African countries and Austria that would allow these immigrants a free entry. Still, as health workers from direct neighbouring countries are actively recruited for Austria, there remains much to consider and scrutinize: for example, in neighbouring Slovakia, the Czech Republic, and Germany, the health care situation is also compromised by a lack of human resources [47,49].

Among other possible reasons for the low number of migrant health workers from sub-Saharan Africa in Austria, one is that the Austrian monarchy and its successors had no colonial ties to Africa. Other studies have traced migration pathways to a historical background and in particular to French and British colonialism [50-52]. In some cases, migrant health workers themselves cite this as a reason to migrate to particular countries in Europe [41]. Nevertheless, this is certainly not the only valid explanation for the current phenomenon of health worker migration [17].

The need to learn the new language, German, in order to be able to work as a health worker in Austria was experienced as a great challenge. On the other hand, in retrospect, it was also viewed as a great achievement. As mentioned, apart from in Namibia, German has not been a widely used colonial lingua franca, in contrast to French or English. Therefore, coming to Austria most migrants have to deal with learning a new language. It is safe to say that migration is particularly challenging for all health workers who migrate to a country where they do not already speak the language [35]. In Austria, health workers have to prove their German language skills in order to obtain accreditation. In addition to the complicated resident and work permit system in Austria, this language barrier can be seen as one important reason that Austria is not an explicit target country for health workers from non-EU member countries.

For most of our study participants, the decision to migrate was situation-dependent rather than primarily professionally motivated. All of our study participants describe difficulties in the health care system in their home country; their testimonies are well reflected in many publications which describe push factors, such as low compensation and bad working conditions, moral distress, or bad education facilities [7,53-55]. Our study participants described political, educational, professional, and social issues in their respective home countries. In Austria, they expected and found better job opportunities, and they also found better educational possibilities. Only in retrospect did several study participants view the working conditions more critically, as we discuss below.

Nevertheless, such push factors came into play only for two of our study participants: the explicit reason to leave for one was that he had trouble finding a good job – otherwise he would have stayed (P 9). The other one found the educational possibilities in his home country too limited (P 7). Lack of opportunity is an important motivational factor for many health workers from sub-Saharan Africa who chose to go abroad [56]. However, migration histories and decisions are often multifactorial and not dependent on professional reasons alone. In addition, migration flows are dependent on socioeconomic factors present in source and destination countries [57]. The same finding is true for the Austrian context: for 7 of our 10 study participants, bad working conditions were neither the initial reason for leaving the home country nor the actual reason for coming to Austria. Most of our study participants came because of their familial ties, and/or because a conflict situation in their home country forced them to seek refuge in any country.

The decision to migrate in a conflict situation is clearly not comparable to leaving one’s home country for personal reasons. In addition, other migration decisions might not be personal, although they appear that way. This applies to health workers who have been educated in a facility that trains them specifically for international practice [10]. In such a case, the intention may have been a foregone conclusion. However, we can neither confirm nor deny this in our findings, as to the best of our knowledge none of our respondents has trained in such a facility.

The education system in the country of origin of sub-Saharan health workers is often shaped after the model of the former colonial power [58]. As mentioned, in Austria the accreditation is complicated due to the different systems in tertiary education.

Our participants’ indications of experiencing racism illustrate the overall situation in Austria, which is frequently shaped by racist prejudices and cultural biases towards “people from the global south” in general, and black Africans in particular [59,60]. Unfortunately, racism towards migrant health workers is not only the case in Austria, but also, for instance, in the UK [61].

As the accreditation of African diplomas is difficult, three of our study participants no longer pursue their health profession. This means that even though they could have been health workers, in Austria they cannot be and therefore are not employed to their full potential. Several of our study participants felt that they were not able to fully live up to their potential. They indicated that they have lost their social status and felt morally conflicted that they had been trained to help people and were not allowed to do so. Those who work at least in a related profession considered their new careers to be a positive compromise. This reflects the results of other studies, which show that due to European legislation, migrants often cannot continue their professions [62,63]. The issue of deskilling applies to migrants in Austria in general and not only migrant health workers or immigrants from sub-Saharan Africa [64]. Deskilling often leads to frustration and loss of social status [65]. Especially in female health worker migration, in particular concerning nurses, deskilling is a well-known issue [65-68].

It is possible to make out clear gender differences in the experiences of our study participants: except for one study participant, all male study participants had been single when they first came to Austria. This means that they pursued the accreditation process on their own. Young men appreciated the opportunity to be independent, even though they had to provide for themselves by working in the low-paying sector. In contrast, several of our female study participants had married Austrian citizens before migrating. They stated to have been financially provided for due to their marriage while going through the accreditation process. However, they also felt reduced to being homemakers, and were frustrated because they were not allowed to work in their professions.

## Conclusion

On the one hand, Austria is not an explicit target country for health workers from sub-Saharan Africa. On the other hand, the competencies of those who live and work in Austria are not used fully. This is a loss for Austria and aggravates the fact that there is a shortage of trained and skilled health workers in the respective country of origin in Africa.

The primary reason for this circumstance appears to be the current resident and work permit regulations in Austria, in particular those applying to citizens from African countries. In addition, the accreditation process and the German language are barriers. Most of the study participants may have considered migration due to the work and study conditions in their home countries. However, they are in Austria *per se* because of personal links rather than specifically and actively wanting to come here.
